# Role of Angiopoietin/Tie2 in Critical Illness: Promising Biomarker, Disease Mediator, and Therapeutic Target?

**DOI:** 10.6064/2012/160174

**Published:** 2012-08-21

**Authors:** Alexander Lukasz, Philipp Kümpers, Sascha David

**Affiliations:** ^1^Department of Nephrology & Hypertension, Hannover Medical School, Carl-Neuberg Straße 1, 30625 Hannover, Germany; ^2^Department of Internal Medicine, Rheumatology and Nephrology, University Hospital Münster, 48149 Münster, Germany

## Abstract

Critical illness is a descriptive, broad term for a serious clinical condition that can result from enormously heterogeneous etiologies. A common end feature these patients regularly suffer from is the so-called multiple organ dysfunction syndrome (MODS), often a consequence of organ hypoperfusion and ischemia, coagulopathies, overwhelming inflammatory responses, immune paralysis and mitochondrial dysfunction. Mechanistically, endothelial injury and particularly microvascular leakage is a major step in the pathophysiology of MODS and contributes to its mortality. The angiopoietin (Angpt)/Tie2 system consists of the endothelial tyrosine kinase Tie2 and its 4 circulating ligands (Angpt1-4). The balance between the agonistic ligand “Angpt-1" and the antagonistic one “Angpt-2" regulates baseline endothelial barrier function and its response to injury and is therefore considered a gatekeeper of endothelial activation. This paper provides a systematic overview of the Angpt/Tie2 system with respect to (1) its role as a global biomarker of endothelial activation in critical ill patients, (2) its contribution to MODS pathophysiology as a disease mediator, and last but not least (3) putative therapeutic applications to modify the activation state of Tie2 in mice and men.

## 1. Introduction

Critical illness is a serious condition that can arise from countless etiologies and that requires highly specialized intensive care units (ICU) for adequate treatment. Among the most common ICU admission reasons are: poisoning, trauma, shock (septic, cardiac, and hemorrhagic), major surgery, respiratory failure, and cardiac ischemia and arrhythmia [1]. One common complication of all these conditions is the so-called multiple organ dysfunction syndrome (MODS) that has a devastating mortality rate [2, 3]. The pathophysiology of MODS is very complex and remains unclear [4, 5]. Hypotheses are multifactorial and range from theories as simple as critical organ hypoperfusion with consequent ischemia to more complex processes as an overwhelming inflammatory response and later immune paralysis [6, 7]. Moreover, the disseminated intravascular coagulopathy (DIC) [8] as well as mitochondrial dysfunction [9] have been demonstrated to play a critical part in the pathogenesis of the syndrome. The repeated failure of clinical trials suggests that some fundamental knowledge is lacking in our current understanding in the development of MODS. A major opportunity, therefore, exists to pursue novel insights into this condition with the hope of improving outcomes. 

## 2. Role of Endothelium 

The endothelium pervades every organ and is responsible for a variety of physiological functions [10], such as regulation of oxygen and nutrients transport, blood pressure, coagulation, inflammatory processes, diapedesis, angiogenesis, and volume control between different compartments [11–13]. Despite spatial and temporal heterogeneity in endothelial cell (EC) responses to the noxious stimuli, each of these functions are altered in critical illness [14, 15]. The net result is that the activated endothelium presents a procoagulant, proadhesive surface; fails to produce its usual profile of vasoconstrictive and vasodilatory compounds; and suffers a loss of normal barrier function. Of these changes, increased vascular permeability may be particularly important because it gives rise to hypovolemia and contributes to hemoconcentration, stasis of blood flow, and shock [16, 17]. In every organ, edema basically increases the distance essential nutrients and oxygen must travel to reach their target cells. Thus, systemic vascular changes have severe consequences for organ function. 

On the molecular level vascular leakage is determined by a combination of disassembled junctional complexes (e.g., vascular endothelial (VE)-cadherin), and myosin driven cell contraction, resulting in paracellular gap formation [11, 14].

The clinically relevant consequences of this so-called “systemic capillary leakage syndrome” are profound decrease in systemic vascular tone, collapse of the microcirculation, and subsequent distributive shock, acute pulmonary distress syndrome (ARDS), and eventually multiple organ dysfunction syndrome (MODS) [14, 18–20]. Thus, the “activated” endothelium might play a crucial role in the pathophysiology of MODS [14].

## 3. The Angiopoietin/Tie2 System

**Figure 1:**
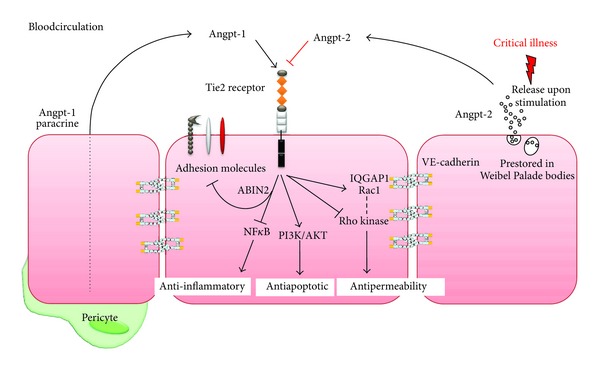
Scheme of the endothelial angiopoietin (Angpt)/Tie2 system highlighting fundamental signalling pathways. Angpt-1 ligation phosphorylates and thereby activates the Tie2 receptor promoting an anti-inflammatory signal via inhibition of surface adhesion molecule expression and the transcription factor NfkB. Moreover, the PI3K/Akt pathway promotes an antiapoptotic, prosurvival signal. The antipermeability effects are induced by maintaining the cytoskeletal architecture in a cortical quiescent formation. On a signalling level this is achieved by simultaneous inhibition of the Rho kinase and activation of the small GTPase Rac1 via IQGAP1 binding. Direct effects on the adherens junction protein VE-cadherin via src have also been reported. Upon stimulation, endothelial cells release prestored Angpt-2 from Weibel Palade bodies into the circulation. Angpt-2 competitively antagonizes positive Angpt-1/Tie2 signalling, thus dramatically activating the endothelial cell and priming it for further inflammatory stimuli.

In 1996/1997, Davies et al. discovered the angiopoietin (Angpt)/Tie2 ligand-receptor system as the second class of transmembrane vascular-specific receptor tyrosine kinases (the first being the vascular endothelial growth factor (VEGF)/VEGF-receptor system) [21]. Gaining attention as an important regulator in vessel maturation and remodeling, several studies demonstrated that the Angpt-Tie2 system not only regulates angiogenesis, but also controls endothelial inflammation and permeability in a nonredundant manner [22, 23]. Ligation of Angpt-1—secreted by pericytes and vascular smooth muscle cells—to the extracellular fibrinogen-like domain of the Tie2 receptor, which is almost exclusively expressed on endothelial cells (and some monocytes) [24, 25] leads to phosphorylation of the intracellular tyrosine split domain of the receptors that promotes endothelial-cell migration and survival mainly via the canonical phosphatidyl-inositol 3-kinase (PI3 K)/Akt pathway [26]. Furthermore, Angpt-1 signaling strongly activates Rac1 via the IQ motif-containing GTPase activating protein 1 (IQGAP1) [27] thereby shifting the RhoA-Rac1 balance towards Rac1, which morphologically results in a cortical F-actin formation promoting structural integrity of the cytoskeletal architecture [28]. Anti-inflammatory properties of Tie2 signaling are promoted via inhibition of nuclear factor kappa-light-chain-enhancer of activated B cells (NF-kB) mediated expression of leucocyte adhesion molecules expression like intercellular adhesion molecule-1 (ICAM-1) and vascular cell adhesion molecule-1 (VCAM-1) [29]. Hence, Angpt-1 mediated Tie2 phosphorylation has anti-apoptotic, anti-inflammatory and antipermeability effects and maintains a quiescent status quo of the endothelium (Figure 1).

 Consistent with the properties of a classical competitive antagonism, Angpt-2 binding to the shared extracellular Tie2 binding domain results in Tie2 dephosphorylation [23]. Data from genetically modified mice underline the hypothesis of functional antagonistic effects of Angpt-1 and Angpt-2 to the Tie2 receptor. Both, Angpt-1-/and Tie2-deficient mice have a lethal phenotype [30, 31]. Consistently, Angpt-2 transgenic mice phenocopy the lethality due to vascular defects like Angpt-1 −/− and Tie2 −/− mice, which suggests that Angpt-2 is indeed a nonredundant antagonistic ligand of Tie2 [32].

 Angpt-2 is stored and rapidly released from endothelial vesicles, the Weibel-Palade bodies (WPB), upon different inflammatory stimuli (e.g., TNF-*α*, LPS, hypoxia) [33]. Angpt-2 sensitizes EC to activation by inflammatory cytokines like TNF-*α* [34]. In Angpt-2 deficient mice leucocytes cannot attach to the endothelium because of a negative NFkB regulation of leukocyte adhesion molecules, which may result from the vast Angpt-1 signaling [32, 35]. Angpt-2 signaling disrupts the integrity of the endothelial barrier through a RhoA mediated endothelial cell contraction and consequent gap formation [23, 36]. Summarized Angpt-2 signaling activates the endothelial layer from a resting state to a proinflammatory state including expression of leukocyte adhesion molecules and a loss of vascular integrity (Figure 1). 

## 4. Angiopoietin-2 as a Biomarker and Putative Mediator of Endothelial Activation 

So far, several groups including our own have investigated circulating Angpt-1 and Angpt-2 levels in critically ill patients. In humans, the balance between both Tie2 ligands appears to be dramatically shifted in favor of Angpt-2, which at least theoretically means that Tie2 receptor signaling is impaired. In sepsis, those Angpt-2 elevations correlate with the severity of illness as assessed by injury severity score [37], organ failure index [38], physiology and chronic health evaluation II (APACHE II) score and sequential organ failure assessment (SOFA) scores [39–44]. 

It all started in 2006 when Dr. Parikh and his coworkers presented the first clinical and experimental study investigating the role of circulating Angpt-2 in ICU patients. Their investigation was based on previous findings that disrupted Tie2 signaling could arise in the inflammatory milieu of sepsis [45, 46], which might predominantly occur in the lung because of high Tie2 expression levels [47, 48]. Therefore, they hypothesized that excessive Angpt-2 release resulting in a competitive Tie2 antagonism may occur in sepsis and promote pulmonary vascular leakage. Besides quantifying circulating Angpt-2 in enrolled patients with mild and severe sepsis, they tested the effect of human septic serum on the morphology of EC architecture *in vitro*. In fact they found that circulating Angpt-2 was elevated in septic patients and associated with oxygenation failure indicated by a PaO_2_/FiO_2_ ratio below 200 (defined as ARDS). In contrast to following studies with a larger number of patients Angpt-2 did not correlate with the APACHE II score, although there was a trend to higher Angpt-2 levels in patients with an APACHE II score >25. *In vitro* experiments with EC showed for the first time that cells incubated with septic patient serum induced actin stress fibers and intercellular gap formation. Recombinant Angpt-2 alone was sufficient to reproduce their earlier findings with septic serum. They concluded that Tie2 signaling could be impaired as a consequence of excess circulating Angpt-2, which leads to Rho-kinase activation and MLC-p, with the end result being EC contraction, gap formation, and disruption of barrier integrity [36, 49]. This landmark paper was the starting shot for different groups evaluating angiopoietins as biomarkers in sepsis around the globe.

Our own group quantified Angpt-2 in septic ICU patients on admission to a medical ICU. All patients had an invasive hemodynamic monitoring which was performed by the pulse contour continuous cardiac output (PiCCO, Pulsion, Munich, Germany) system to test the hypothesis if high Angpt-2 levels are associated with hemodynamic parameters and surrogates of vascular leakage like the extravascular lung water index (EVLWI). In line with previous reports Angpt-2 concentrations were increasingly higher across strata of the sepsis syndrome (i.e., healthy controls, ICU patients without sepsis, patients with sepsis, and patients with septic shock). Furthermore, Angpt-2 correlated with surrogate markers of pulmonary dysfunction (PaO_2_/FiO_2_) and tissue hypoxia (serum lactate). Surprisingly, there was no association between Angpt-2 and any hemodynamic parameter including EVLWI.

 Likewise van der Heijden et al. detected no association between EVLWI and Angpt-2, which may show the limitation of this method to detect permeability in mechanically ventilated patients. Assessed with the ^67^Gallium-labelled transferrin method van der Heijden et al. found a correlation between the pulmonary leak index and circulating Angpt-2 [44]. In a multivariate regression model Angpt-2 admission levels outperformed the SOFA and APACHE II score and was an independent marker of 30-day survival [41]. 

To this point, it was unclear if elevation of circulating Angpt-2 is just an epiphenomena of otherwise activated endothelium (e.g., interleukins) or if it is indeed a direct consequence of the septic stimuli. However, our observation that healthy volunteers subjected to a very small dose of LPS led to a dramatic increase of circulating Angpt-2 within less than 2 hours, even preceding the release of some traditional proinflammatory cytokines, makes a direct endothelial Angpt-2 release upon LPS stimulation likely [51]. 

Recently published large clinical trials provide further evidence for Angpt-2's potential as a promising biomarker of severity and mortality prediction in a slightly different cohort of patients with acute lung injury (ALI) [52–54]. Calfee and colleagues included an impressive 931 patients with ALI of infectious and noninfectious origin in their study [52]. In non-infection-related ALI, higher baseline Angpt-2 levels were strongly associated with increased mortality. Confusingly, in infection-related ALI there was no Angpt-2 difference in baseline values between survivors and nonsurvivors. This finding is in contrast with previous findings in which baseline Angpt-2 was consistently associated with outcomes in sepsis (i.e., infection)-related ALI. Calfee and colleagues discuss a spectrum bias or differences to previous studies in timing of plasma samples as a possible reason.

In 2010, Ong and colleagues found in a prospective study that the ratio of Angpt-2 to Angpt-1 did predict mortality in 56 ALI patients whereas other factors of endothelial markers (IL-6, IL-8, vWF, thrombomodulin, protein c, icam-1) did not [54]. Given the Tie2 antagonistic concept of Angpt-1 and Angpt-2 this approach using the ratio between both circulating ligands is particularly interesting and deserves careful further analysis.

Acute liver failure (ALF) is a critical condition associated with a severe inflammatory response regularly resulting in massive vascular leakage. We hypothesized that the Angpt/Tie2 system might be involved in this process. Indeed, we observed that Angpt-2 levels in patients with noninfection related primary ALF were associated with the severity of illness. Patients with later transplant free recovery had lower levels of Angpt-2 as those who reached the composite endpoint of death or emergency liver transplantation. Even more interesting, Angpt-2 was identified as an independent predictor of this composite end point. *In vitro* studies underlines the hypothesis of Angpt-2 release from the liver [55]. 

Together, these results from various clinical and experimental studies demonstrate the potential of Angpt-2 as an endothelial biomarker for severity of illness and outcome prediction in critically ill patients. However, larger prospective trials (in particular in sepsis) are highly desirable to establish Angpt-2 measurements for clinical use. 

## 5. Manipulating the Tie2 Receptor as a Putative Treatment Strategy

### 5.1. The Ligand Perspective

In 2005, Witzenbichler et al. demonstrated in a fascinating *in vivo* experiment the protective role of Angpt-1 in lipopolysaccharide (LPS) induced endotoxic shock. As a vector to achieve high levels of circulating Angpt-1 they used an adenoviral construct expressing either the human Angpt-1 protein (AdAngpt-1) or a green fluorescent protein (AdGFP) as a control treatment. Hemodynamic function assessed by left heart catheterization was severely depressed in AdGFP compared to AdAngpt-1 treated mice. In detail, mean arterial blood pressure, contractile ability of the heart (initial velocity − *dP*/*dt* max), and cardiac output were lower in control mice. Lung water content and activity of pulmonary myeloperoxidase activity as parameters of capillary leakage and lung inflammation were also reduced in the AdAngpt-1 group. The LPS-induced increase in expression levels of pulmonary VCAM-1, ICAM-1, and E-selectin as a marker of endothelial inflammation was markedly lower in AdAngpt-1 mice than in control mice. Furthermore, the survival of AdAngpt-1 mice was slightly superior to control mice (30% overall survival benefit). These experiments showed improved hemodynamic function, reduced lung injury, and a lower inflammatory response accompanied by an improved survival rate in AdAngpt-1 treated mice. Although, adenoviral transduction is generally not feasible in humans and leads to a very high expression of Angpt-1 (up to 1 ug/mL) the authors demonstrated here a potential role of Angpt-1 as an adjunctive agent for the treatment of septic shock [56] and opened a new avenue for specific sepsis therapeutics. 

 In 2007, Mei et al. provided further evidence for the protective potential of Angpt-1. They showed in a set of *in vivo* experiment the effect of mesenchymal stem cells (MSC) overexpressing Angpt-1 for the prevention of LPS-induced ALI in mice. MSC with or without transfection with the plasmid containing the human Angpt-1 gene (pAngpt-1) were injected in the mice after intratracheal instillation of LPS to induce ALI. Both groups showed a dramatic reduction in LPS-induced pulmonary inflammation quantified by neutrophils and cytokines in the bronchoalveolar space. Of note, the administration of MSC transfected with pAngpt-1 resulted in a nearly complete reversal of LPS-induced pulmonary hyperpermeability, as reflected by reductions in IgM and albumin levels in bronchoalveolar lavage (BAL) [57]. These results underline the potential of a cell-based Angpt-1 gene therapy in the treatment of ALI/ARDS. 

In sum, a benefit of upregulating Angpt-1 by different means *in vivo* has been shown in the context of endotoxic shock on the one hand and ALI/ARDS on the other hand. Unfortunately, these approaches using adenoviral gene transfer or modified-stem cell delivery are not feasible to translate in humans yet. 

 In 2009, Kim et al. determined the positive effect of an increased Angpt-1 signaling in LPS-induced acute kidney injury using a slightly different approach. Mice were pretreated with an engineered variant of native Angpt-1, the so-called Angpt-1 with cartilage oligomeric matrix protein (COMP Angpt-1), which is more potent in phosphorylating Tie2 than native Angpt-1. Three days after COMP Angpt-1 pretreatment mice were challenged with LPS. LPS-induced acute kidney injury (AKI) was ameliorated in the COMP angpt1 group possibly caused by improved renal and systemic hemodynamics, as reflected by an increase of renal blood flow and mean arterial pressure. The inulin-measured glomerular filtration rate was significantly higher in the treated group than in the control group. Again, decreased renal ICAM-1 and VCAM-1 protein expression levels were detected. Furthermore, macrophage infiltration was decreased in COMP Angpt-1 pretreated mice [58]. These results show the protective effect of Angpt-1 signaling, in this case with an engineered variation, COMP Angpt-1, against endotoxic AKI. A comparable experiment with a rescue application of COMP Angpt-1 after LPS infusion to simulate a more realistic setting was not provided but would be highly desirable. 

Another way to therapeutically modulate Tie2 phosphorylation is the administration of a PEGylated 7-mer, HHHRHSF, which was recently identified by screening a phage display library for binding to the Tie2 receptor [59]. A polyethylene-oxide clustered version of this peptide is called Vasculotide (VT) and activates Tie2 very effectively and for over 72 hours [60]. In human microvascular endothelial cells (HMVECs), we could show that VT prevented morphological endotoxin-induced gap formation, functional loss of monolayer resistance, and translocation of labeled albumin. These in vitro findings are comparable to Parikh's work from 2006, who showed an analogous protective effect of recombinant Angpt-1 for endothelial cell integrity [36, 50]. 

**Figure 2:**
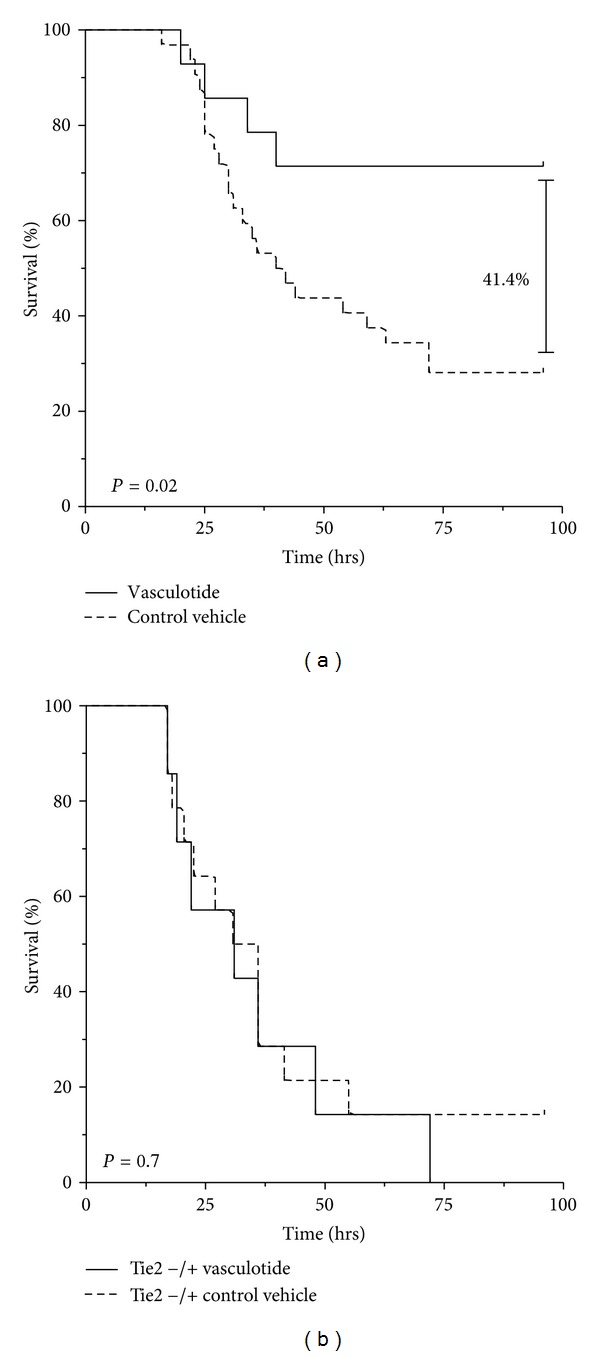
Tie2 activation via the synthetic pegylated 7-mer “HHHRHSF” termed Vasculotide (VT) improves ssurvival in experimental murine sepis. (a) C57BL6 mice were pretreated with 500 ng VT (*n* = 15) or PBS (control, *n* = 30) 7 h prior to injection of a 70% lethal LPS dose. VT improved survival by 41.4% (*P* = 0.02). (b) Male Tie-2 heterozygous (Tie-2 +/−) mice were pretreated with 500 ng VT or PBS 7 h prior to injection of LPS. Survival was indistinguishable (*P* = 0.66) excluding unspecific off target effects of VT. (Modified after: [50].)

From a pharmaceutical point of view VT may hold promise as a drug-like compound. We therefore assessed VT *in vivo* in a model of endotoxemic ALI. And indeed, a single VT injection 7 hours prior to LPS was sufficient to prevent the development of lung vascular leakage, as demonstrated by Evans blue extravasation. Lungs of mice treated with LPS and an empty PEGylated backbone had a 4-fold higher increase in dye extravasation than those treated with VT. VT pretreatment also improved survival of endotoxemia by 41% an effect that was completely abolished in Tie2 heterozygous knockout mice (Figure 2). Indicating the high specificity of VT for Tie2. Rescue experiment with VT given 2 hrs after LPS administration showed only a trend towards improved survival by 33%. Furthermore, echocardiographic evaluations showed also a nonsignificant trend toward improved myocardial performance associated with VT [50]. This finding is discrepant to Witzenbichler's who found improved cardiac performance but might be explained by the differences between Angpt-1 and VT. Again, VT is a 7-mer that specifically (and probably exclusively) binds Tie2. At least in vitro, Suzan Dallabrida demonstrated in 2005 that native, full-length Angpt-1 could also bind integrins on cardiomyocytes thereby improving contractility of this cell type [61]. 

In another study of our own group Kumpers et al. also provided evidence for the high therapeutic potential of VT in the context of polymicrobial abdominal murine sepsis. Their key finding was that prophylactic as well as therapeutic (i.e., rescue) administration of VT was sufficient to reduce mortality in a clinically relevant experimental murine sepsis model [62]. Both reports provide a proof of principle for the potential use of the Tie2 receptor agonist, VT, as a strategy against vascular leakage thereby improving organ function and survival rates in 2 different models of murine sepsis. 

### 5.2. Perspective of the Antagonist

Given that circulating levels of Angpt-2 (the natural antagonist of Tie2) were so high in different septic clinical populations the question arises if Angpt-2 directly contributes to sepsis severity rather than “just” being a marker of severity. In fact, this question has been asked for a long time but never been answered satisfactory in the “*vascular biology-sepsis community*.” 

The first proof of principle Tie2 loss-of-function experiment has been provided by Parikh and coworkers in 2006. Adult mice were pretreated via intraperitoneally injection with a relatively high dose of Angpt-2 or vehicle. The miles assay, an Evans blue extravasation technique, was then used to quantify the vascular leakage. The extravasated dye (that covalently binds to albumin) showed enhanced leakage with a 3-fold increase in lungs and 2-fold increase in livers of Angpt-2 challenged mice compared with vehicle treated controls [36]. These results were the first *in vivo* to demonstrate a putative mechanistic role of Angpt-2 in disrupting the integrity of the endothelium. 

Fiedler et al. used an Angpt-2 deficient (−/−) mouse to analyze leucocyte-endothelial interaction in a sterile peritonitis model by intravital microscopy. They could convincingly demonstrate that Angpt-2 deficiency leads to a relevant reduction in adhesion molecules and a consequent failure to firmly adhere to leucocytes [34]. Furthermore, Bhandari et al. found in the same global Angpt-2 knockout mouse that hyperoxia induced ALI is an Angpt-2 driven process that can be ameliorated in the absence of Angpt-2 [63]. 

Besides these reports so far no data have been published to ultimately prove a mechanistic role of Angpt-2 in the development of MODS in clinically relevant sepsis models.

We therefore designed a series of experiments to investigate the role of Angpt-2 in sepsis morbidity and mortality. In fact, Angpt-2-deficient mice have less organ failure and a survival benefit exceeding 40% in two different murine sepsis models (endotoxin and polymicrobial sepsis) most likely by ameliorating the critical Angpt-2-driven Tie2 deactivation [64]. Moreover, severe morphological changes induced in EC culture by coincubation with serum from septic patients where completely reversible after treatment with an Angpt-2 function-blocking antibody. These *in vivo* and in vitro experiments provide evidence that Angpt-2 directly contributes to the adverse outcomes in sepsis.

A very recent PNAS study from Ghosh et al. comprehensively demonstrated the potential of any Tie2 modulating therapy in another model of critical illness, that is, lethal anthrax infection. Clinically, anthrax infection is dreaded for its dramatic vascular leakage. Therefore, the authors hypothesized that the Angpt/Tie2 system might be involved in this system and found upregulated Angpt-2 in murine and primate models of the disease. They used 2 different knockout models and an Angpt-2 delivering adenovirus (AdAngpt-1) to prove the mechanistic involvement of Angpt/Tie2 in this condition. Interestingly, not only AdAngpt-1, but also Angpt-2 deficient mice showed improved survival in an otherwise 100% lethal anthrax model. Again, as a proof of principle Tie2 deficient mice had a worse outcome using a lower dose of the toxin [65].

## 6. Conclusion and Future Perspectives

Within the last 5 years, several studies around the globe established the role of elevated circulating Angpt-2 as a biomarker of severity of illness and outcome prediction. Although a promising candidate of global endothelial activation Angpt-2 is not ready for prime time yet. To justify the use of circulating Angpt-2 as an additional routine diagnostic and/or prognostic test large prospective clinical trials are required. In this context, the role of the angiopoietin balance has been given little less attention although theoretically this balance appears to be more important than the absolute levels. 

The fundamental role of Tie2 activation as a consequence of alterations in the competitive antagonists Angpt-1 and Angpt-2 in various experimental models of critical illness has been demonstrated by independent groups. Angpt-2 appears to be a disease mediator directly contributing to morbidity and mortality. 

Different Tie2 manipulating experimental tools from antibodies to agonistic peptides are available but have not been tested in men. A series of experimental data suggest that both an exogenous Tie2 activating as well as an Angpt-2 inhibition strategy might not only be effective in mice but also generally feasible in men. It is well possible that inhibition of a natural inhibitor (Angpt-2) could reasonably be expected to restore Tie-2 phosphorylation to pre-sepsis levels whereas exogenous receptor activation offers greater titration control, even enabling one to exceed quiescent levels of Tie-2 activation.

In spite of removing activated protein C as the first (and only) specific endothelial targeting sepsis therapeutic from the market, other potential targets make the vasculature a promising target.
